# Recent advances in understanding regulation of the Arabidopsis circadian clock by local cellular environment

**DOI:** 10.12688/f1000research.21307.1

**Published:** 2020-01-27

**Authors:** Timothy J. Hearn, Alex A.R. Webb

**Affiliations:** 1 Department of Plant Sciences, University of Cambridge, Downing Site, Cambridge, CB2 3EA, UK; 2 Research Department of Cell and Developmental Biology, Rockefeller Building, University College London, London, WC1E 6DE, UK; 3 Academic Department of Medical Genetics, University of Cambridge, Cambridge Biomedical Campus, Cambridge, CB2 0QQ, UK

**Keywords:** Circadian, dynamic, cellular, Arabidopsis, regulator

## Abstract

Circadian clocks have evolved to synchronise an organism’s physiology with the environmental rhythms driven by the Earth’s rotation on its axis. Over the past two decades, many of the genetic components of the *Arabidopsis thaliana* circadian oscillator have been identified. The interactions between these components have been formulized into mathematical models that describe the transcriptional translational feedback loops of the oscillator. More recently, focus has turned to the regulation and functions of the circadian clock. These studies have shown that the system dynamically responds to environmental signals and small molecules. We describe advances that have been made in discovering the cellular mechanisms by which signals regulate the circadian oscillator of Arabidopsis in the context of tissue-specific regulation.

## Introduction

A circadian oscillator is a biological system that generates free-running rhythms of near 24 h. The period probably arises from delays in the oscillator network rather than network structure, and regulatory events might be needed to keep the period close to 24 h. Because the system is subject to regulation, plant circadian period is not fixed but rather is variable depending on conditions ^1^. The ability of the circadian oscillator to respond to signals has been termed “dynamic plasticity” because period is plastic and the degree of plasticity changes throughout the day ^1^. Whilst it is convenient to measure circadian period in constant conditions, the evolutionary importance of changes in circadian period has probably arisen to ensure the correct timing of events in light and dark cycles. Dynamic plasticity might allow the circadian system to adjust to cues from the rhythmic environment to ensure the correct entrained phase, which is the time of cellular events with respect to the environment. This ensures that internal events are timed appropriately (for example, that internal dawn matches external dawn). Entrainment also ensures that the circadian oscillator can track the change of time of dawn and dusk which occurs throughout the seasons in higher latitudes. Because the day and night lengths vary throughout the seasons, the relative phase of these internal dawn and dusk events changes with respect to each other and also with the phase of other components in the oscillator as a result of dynamic plasticity. We describe the small molecules and environmental signals that have recently been demonstrated to alter circadian period; where known, we outline the mechanisms that regulate the circadian oscillator to set the period.

## Transcriptional and post-transcriptional mechanisms adjust circadian period in response to environmental changes

Circadian oscillator components oscillate in either abundance or activity under constant conditions, they feedback to regulate the activity of other oscillator components and therefore if the oscillating abundance or activity is clamped to a high steady state this can abolish circadian rhythms. In addition to the Arabidopsis core oscillator components that meet these definitions ^2, 3^, there are genes that are not strictly oscillator components but nevertheless can affect circadian period. These include genes affecting gene regulation and protein stability. For example, RNA splicing is strongly implicated in the regulation of circadian period, and SICKLE (SIC) has a role as a regulator of *CIRCADIAN CLOCK ASSOCIATED 1* ( *CCA1*) and *LATE ELONGATED HYPOCOTYL* ( *LHY*) splice variants, affecting the temperature regulation of the *PSEUDO RESPONSE REGULATOR 7* ( *PRR7*) promoter ^4^. RNA splicing and circadian period are also regulated by the PLANT U-BOX 59 and PLANT U-BOX 60 (MAC3A and MAC3B) proteins demonstrated by an innovative assay for identification of E3 ligases that bind to clock components ^5^. MAC3A and MAC3B are orthologous to the animal E3 ubiquitin ligase Pre-mRNA Processing factor 19 (Prp19). E3 ligases have a central role in the oscillator, and ZEITLUPE (ZTL) acts as an E3 ligase that directs degradation of TIMING OF CAB EXPRESSION 1 (TOC1) and PRR5, resulting in a long circadian period in *ZTL* loss-of-function plants ^6^. By contrast, mutants in the deubiquitinases UBIQUITIN-SPECIFIC PROTEASE 12 (UBP12) and UBP13 have a short circadian period ^7^ through effects on ZTL ^8^. *BIG*, a gene with homology to the mammalian E3 ligase *UBR4*, regulates circadian period in a photoperiod-dependent manner ^9^, but, to our knowledge, BIG has not been demonstrated to have functional E3 activity.

In addition to ubiquitination, the SMALL UBUIQUITIN-LIKE MODIFIER (SUMO) proteins regulate circadian period through post-translational modification of target proteins. Dependent on temperature, increased global cellular SUMOylation increases circadian period whereas decreased SUMOylation reduces period ^10^. This temperature dependence has led to the suggestion that SUMOylation participates in buffering the oscillator against changes in temperature. Whereas ubiquitination affects protein stability, SUMOylation might affect function because increased SUMOylation of CCA1 reduced its affinity for the promoters of target genes ^11^.

Changes in circadian period in response to temperature are not mediated only by SUMOylation; several genes associated with different biological processes have been associated with the effects of temperature on circadian period. HEAT SHOCK PROTEIN 90 (HSP90) is induced in response to high temperature stress to regulate the circadian oscillator through a GIGANTEA (GI)-dependent mechanism ^12^. HSP90 also has a GI-independent effect in regulating circadian period and has a circadian phenotype that is greater in seedlings entrained in hot–cold cycles and a phase shift caused by warmth in the morning ^13^. *COLD-REGULATED GENE27* and *28* ( *COR27/28*), ** which are typically associated with the cold response of Arabidopsis, are required for low temperature-dependent regulation of circadian period. *cor27/28* mutants have a long period in blue light and low temperature ^14^. *COR27/28* act as night-time repressors of *PRR5* and *EARLY FLOWERING 4* ( *ELF4*) and are regulated by CCA1 and, in turn, bind to the *TOC1* and *PRR5* promoters. COR27 and COR28 are required for the functions of PRR7 and PRR9 in entrainment, suggesting a role for COR27 and COR28 in temperature entrainment of the circadian clock ^15^.

The *JUMONJI DOMAIN CONTAINING 5* (JMJD5) histone demethylase contributes to temperature compensation ^16^ but does not directly methylate histones at circadian *loci*, indicating another role for this potential demethylase protein within the circadian system.

## The endogenous circadian and cell cycle oscillators are coupled

In addition to the new insights concerning the integration of the circadian clock with environmental signals, the oscillator has recently been discovered to be associated with the endogenous cell cycle. TOC1 is required for the G _1_–S transition in leaves ^17^, similar to the interrelationship between Metazoan circadian and cell cycles ^18^. TOC1 regulates CDC6 and the DNA pre-replicative machinery to ensure that growth is resonant with the environment. A slow-running circadian oscillator causes a slower progression through the cell cycle, and vice versa, potentially indicating coupling rather than gating ^17^. Possibly related to the link between plant cell and circadian cycles is the recent finding that repair of ultraviolet light–induced lesions in DNA is modulated by the circadian clock ^19^, contributing to between 10 and 30% of transcription-coupled DNA repair.

## Small molecules and hormones are regulators of circadian period

Metabolites, hormones and ions are also endogenous regulators of circadian period ^1^. The plant hormones abscisic acid (ABA), ethylene, jasmonic acid (JA) and salicylic acid (SA) all affect circadian period, seeming to act through different pathways. Ethylene reduces circadian period through a pathway that involves GI ^20^. SA conversely slightly increases period and causes strong phase delays following transient stimulation ^21^. These delays are reduced in *NONEXPRESSER OF PATHOGENESIS-RELATED* *GENES 1* ( *NPR1*) mutants, indicating that the effect is mediated by this common SA transcription factor. Exogenous JA-isoleucine, a bioactive form of JA, also increases circadian period ^22^. The response to JA seems to be mediated through the canonical JA signalling pathway requiring the JA receptor COI1 ^22^. Exogenous application of ABA reduces circadian period dependent upon *PRR7* ^23^. ABA signalling to the oscillator involves MYB96, which regulates the gating of ABA responses, and *TOC1* is required for the correct induction of some ABA-responsive genes ^24^. *TOC1* also participates in the regulation of circadian period by changes in the cytosolic-free Ca ^2+^ concentration, which was demonstrated by the epistasis of *TOC1* mutants with mutations in the *CALMODULIN-LIKE 24* gene that encodes a Ca ^2+^ sensor ^25^.

Sucrose sustains circadian oscillations in continuous dark through stabilisation of the GI protein, which also inhibits effects of ethylene ^20^. Exogenous sugars also reduce circadian period in plants that have had their internal levels of sugars lowered by low light or inhibition of photosynthesis ^26^. A short pulse of exogenous sucrose advances circadian phase in the early photoperiod because period and phase are related aspects of oscillator function ^1, 26, 27^. Loss of function of either the early morning–expressed *CCA1* or the later-expressed *PRR7* renders circadian period insensitive to sugars ^26, 28^. In a mathematical simulation, the response of the circadian oscillator to sugars can be explained by a simple loop involving an early-expressed gene activator (representing *CCA1*) and a later-expressed repressor (representing *PRR7*) ^29^. The first transcriptional response to low sugars is an increase in *PRR7* transcript abundance leading to the proposal of *PRR7* as an entry point for sugar signalling in the circadian system ^26^. The sugar status–sensitive transcription factor BZIP63 binds and regulates *PRR7* to change phase in response to sugars ^27^. Genetic data suggest that trehalose 6 phosphate (T6P) is the signalling sugar that reports sugar status to the oscillator ^27^, and it has been proposed that regulation of SNrK1 kinase activity by T6P controls the binding/activity of bZIP63 at the *PRR7* promoter. Whether the regulation of *CCA1* by sugars is through PRR7 ^26^ or more directly through the PHYTOCHROME INTERACTING FACTORS (PIFs) ^30^ will be resolved through further experimental testing, which will also establish whether transcriptional changes in either *CCA1* or *PRR7* are sufficient to explain the changes in circadian period and phase.

Sugars and light signalling can also affect the timing of outputs of the oscillator. For example, the clock- and energy-regulated promoter of *DARK-INDUCED 6* has peak activity at night but in constant light this shifts to subjective day ^27^, whereas the phosphorylation of RIBOSOMAL PROTEIN S6 (RPS6) normally peaks in the day but at subjective night in constant light ^31^. In both cases, sucrose added to the media interferes with the different light and clock signals; as a result, the timing of the peak is the same in light–dark cycles and constant conditions.

It has been proposed that sugars entrain the oscillator to set the clock to a “metabolic dawn” as an adjustment to changes in photosynthate production caused by altered light intensity ^26^ or it might contribute to the regulation of carbon homeostasis by regulating transitory starch reserves ^32^. Alternatively, the regulation by sugars is a form of retrograde signalling from the plastid to the nucleus ^33^. A plastid-based signal might be found in the diurnal accumulation of tetrapyrrole, the core molecule of chlorophyll to link plastid signalling to cold signalling ^34^. This signal is proposed to inhibit HSP90, the chaperone that stablises ZTL. This inhibition leads to an increase in expression of *ELONGATED HYPOCOTYL 5* ( *HY5*) and *PRR5* which repress C-REPEAT BINDING FACTORS (CBFs), giving a mechanism for loss of downstream cold-responsive gene expression during the photoperiod.

The ability of the circadian oscillator to change period might be associated with the function of *PRR7.* Loss of function of PRR7 renders the circadian oscillator insensitive to sucrose and nicotinamide ^35^ and more responsive to ABA ^23^. Nicotinamide increases circadian period through inhibition of Ca ^2+^ signalling in a blue light–dependent manner ^35^. *PRR7* is not essential for the response to nicotinamide since plants in which both *PRR7* and *PRR9* are lost are hyper-responsive. Systems identification and a new modelling approach that pinpoints the areas of a system which are being perturbed suggested that the regulation between PRR7 and PRR9 and the activity of TOC1 might be important for changes in circadian period in response to nicotinamide ^35^.

## Photoperiod is a regulator of circadian period

As photoperiod lengthens (and conversely the skotoperiod decreases), there is an increase in the period of the Arabidopsis circadian oscillator ^9^. This is an example of so-called aftereffects in which the free-running period is affected by the prior entrainment conditions ^36^. It is likely that aftereffects represent a change in oscillator behaviour that occurs to regulate the phase relationship between the internal oscillator and the light–dark cycle in different seasons to ensure the correct timing of events in different photoperiods by integration of circadian and light signalling ^37^. The mechanisms by which the oscillator changes its timing in response to seasonal changes might include *BIG* ^9^. The regulation of growth in different photoperiods might involve the binding of TOC1 to PIFs to stop activation of growth until pre-dawn in short days ^38^. The activity of PIFs to dawn is also gated by binding of both PIFs and PRRs to G boxes in target promoters ^39^.

PIFs might be associated with responses to photoperiod because they affect the pace of the circadian oscillator ^30, 40^. Under high fluence rate of light, a *PIF1,3,4,5* quadruple mutant has a longer circadian period than wild-type plants and the various over-expressor lines have a slightly shorter period ^30^. The effect of PIFs on circadian period is dependent on the concentration of sucrose in the media ^30^. HY5, a transcription factor that acts downstream of blue light signalling, also seems to be involved in the regulation of circadian period because *hy5* mutants have short free-running circadian rhythms in monochromatic blue light but not red or in darkness ^41^.

The regulation of clock gene expression by PIFs and other regulators is an example of how the cell might be entrained to photoperiod parametrically through light signalling, but circadian phototransduction might also be integrated from the nucleus to the chloroplast. First, there is the report that PHOTOTROPIN mutants do not affect clock gene expression in either the morning or evening complexes ^42^ but do affect circadian photosynthetic rhythms, indicating a possible role in transducing circadian light signals to the chloroplast. Second, it is found that the chloroplast transcription response to light mediated by SIGMA FACTOR 5 (SIG5) integrates circadian phototransduction with chloroplast transcription by relaying information on blue light dependent upon CRYPTOCHROME ^43^.

The correct alignment of circadian time to the external environmental rhythm provides advantage to the plant ^44^ and therefore it might be expected that changes in photoperiod, with the associated change in circadian timing, have a consequence for the performance of the cell. Evidence for this is provided by “circadian stress” in plants with reduced cytokinin levels or defective cytokinin signalling, which have increased leaf death in response to changes in photoperiod ^45^. It appears that one function of cytokinins is to suppress the stress caused by the changing oscillator period and the relationship between the internal and external time that occurs during photoperiodic transitions.

## Organ- and cell-specific regulation of circadian period

The plasticity of the circadian oscillator in response to light, hormones and metabolites might predict that the circadian clock functions differently in different cell types and tissues. There is experimental evidence to support this hypothesis. Imaging of luciferase reporters of promoter activity separated the function of the circadian systems of the roots and shoots ^46^. The oscillators in the roots respond to light more strongly than those in the leaves because of greater sensitivity to red light ^47^. It has been proposed that the roots in the soil are not in total darkness since light is piped through the vasculature to the root cells ^47^. There is also enrichment in the expression of evening-expressed genes in the roots, and mutations in clock genes give organ-specific phenotypes, such as the dampening of the root clock in *gi-2* ^48^.

With high-resolution quantitative time-lapse microscopy of CCA1–YFP fusion proteins, it was possible to detect robust single-cell circadian oscillations *in planta* that become desynchronised in constant conditions because of the oscillators in different cells running at slightly different speeds ^49^. Whilst cells become desynchronised, there is some weak coupling between the oscillators which results in two waves of clock gene expression going up and down the root in constant conditions ^49^. The different period in individual cells might be explained by local cellular conditions, such as the relative levels of hormones or metabolites. The effect of local cellular conditions on the oscillator might explain the different free-running circadian period that is measured in different organs, such as in older leaves in which the oscillator runs faster than in younger tissue ^50^ and the very fast circadian oscillator measured in the root tip ^51^. The adaptive nature of the differential regulation of the oscillator in different tissues is demonstrated by the discovery that oscillator period differs between tissues both in constant conditions and in entraining light and dark cycles and this results in phase differences between different tissues which are explained by models evoking weak local coupling and regulation by tissue-specific environmental conditions ^51^.

## Summary remarks

**Figure 1.**
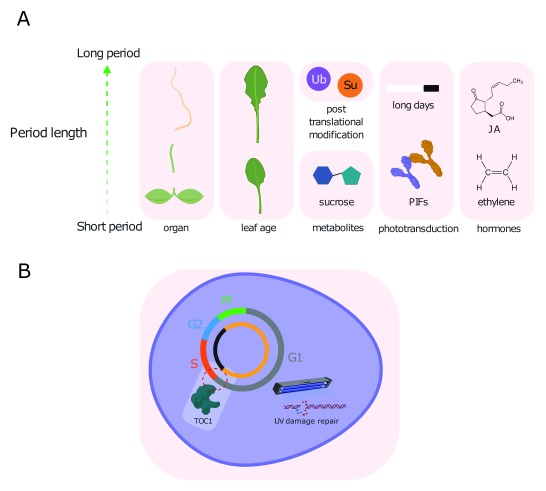
The period of the *Arabidopsis thaliana* circadian clock is regulated extensively by the cellular environment. ( **A**) Cellular factors that affect the free-running period length of the Arabidopsis circadian clock include organ, age, post-translational modifications such as ubiquitination and SUMOylation, metabolites such as sucrose, hormones and phototransduction. ( **B**) In turn, the circadian clock regulates the duration and integrity of the cell cycle, as entry into S phase around dusk is gated by the clock through TOC1, and DNA double-strand break repair occurs during G phase when transcription is active. JA, jasmonic acid; PIF, PHYTOCHROME INTERACTING FACTOR; Su, SUMOylation; TOC1, TIMING OF CAB EXPRESSION 1; Ub, ubiquitin.

The sensitivity of circadian behaviour to cell type and conditions, including osmolarity ^52^, requires that there is careful reporting of the experimental protocols, conditions and data captured. Open science platforms such as Biodare2 ( https://biodare2.ed.ac.uk/) that enable high-quality archiving, analysis and presentation of circadian datasets will facilitate the sharing of protocols and data for analysis between the community ^53^. For much of the past two decades, the focus of plant circadian research has been to identify the components of the oscillator and understand the network structure that generates the oscillatory dynamics. In recent years, a wealth of investigations have demonstrated that the circadian oscillator is sensitive to cellular conditions and this can result in different entrained phases dependent on tissue type ( Figure 1). The future challenge will be to consider which cell types and environmental conditions will be most appropriate for the circadian response to be investigated. Greater focus on cell type–specific responses will help identify the signalling pathways by which signals regulate the oscillator. These studies might provide insight into why the oscillator has such dynamic plasticity and identify the output pathways whose phase is being adjusted and for what purpose.

## Abbreviations

ABA, abscisic acid; CCA1, CIRCADIAN CLOCK ASSOCIATED 1; COR27, COLD-REGULATED GENE 27; GI, GIGANTEA; HSP90, HEAT SHOCK PROTEIN 90; HY5, ELONGATED HYPOCOTYL 5; JA, jasmonic acid; PIF, PHYTOCHROME INTERACTING FACTOR; PRR, PSEDUO RESPONSE REGULATOR; SA, salicylic acid; SUMO, SMALL UBUIQUITIN-LIKE MODIFIER; T6P, trehalose 6 phosphate; TOC1, TIMING OF CAB EXPRESSION 1; ZTL, ZEITLUPE
